# ALD-Derived WO_3–*x*_ Leads to Nearly Wake-Up-Free Ferroelectric Hf_0.5_Zr_0.5_O_2_ at Elevated Temperatures

**DOI:** 10.1021/acsaelm.5c02359

**Published:** 2026-02-04

**Authors:** Nashrah Afroze, Jihoon Choi, Salma Soliman, Chang Hoon Kim, Jiayi Chen, Yu-Hsin Kuo, Mengkun Tian, Chengyang Zhang, Priyankka Gundlapudi Ravikumar, Suman Datta, Andrea Padovani, Jun Hee Lee, Asif Khan

**Affiliations:** † Department of Electrical and Computer Engineering, 1438 Georgia Institute of Technology, Atlanta, Georgia 30332, United States; ‡ School of Energy and Chemical Engineering, 131639 Ulsan National Institute of Science and Technology (UNIST), Ulsan 44919, South Korea; § Institute of Materials and Systems, 115724 Georgia Institute of Technology, Atlanta, Georgia 30332, United States; ∥ Department of Materials Science and Engineering, Georgia Institute of Technology, Atlanta, Georgia 30332, United States; ⊥ Department of Engineering Sciences and Methods (DISMI), University of Modena and Reggio Emilia, Reggio Emilia 42122, Italy; # Graduate School of Semiconductor Materials and Devices Engineering, 9306 Ulsan National Institute of Science and Technology (UNIST), Ulsan 44919, South Korea

**Keywords:** ferroelectric memory, HfO_2_-based ferroelectrics, oxygen reservoir layer, ALD deposited WO_3−*x*_, high-temperature operation, nearly wake-up-free, memory-on-logic, 3D integration

## Abstract

Breaking the memory wall in advanced computing architectures will require complex 3D integration of emerging memory materials such as ferroelectrics—either within the back-end-of-line (BEOL) of CMOS front-end processes or through advanced 3D packaging technologies. Achieving this integration demands that memory materials exhibit high thermal resilience, with the capability to operate reliably at elevated temperatures, such as 125°C, due to the substantial heat generated by front-end transistors. However, silicon-compatible HfO_2_-based ferroelectrics tend to exhibit antiferroelectric-like behavior in this temperature range, accompanied by a more pronounced wake-up effect, posing significant challenges to their thermal reliability. Here, we report that by introducing a thin tungsten oxide (WO_3–*x*_) layer—known as an oxygen reservoir—and carefully tuning its oxygen content, ultrathin Hf_0.5_Zr_0.5_O_2_ (5 nm) films can be made robust against the ferroelectric-to-antiferroelectric transition at elevated temperatures. This approach not only minimizes polarization loss in the pristine state but also effectively suppresses the wake-up effect, reducing the required wake-up cycles from 10^5^ to only 10 at 125°C, a qualifying temperature for back-end memory integrated with front-end logic, as defined by the JEDEC standard. First-principles density functional theory (DFT) calculations reveal that WO_3_ enhances the stability of the ferroelectric orthorhombic phase (o-phase) at elevated temperatures by increasing the tetragonal-to-orthorhombic phase energy gap and promoting favorable phonon mode evolution, thereby supporting o-phase formation under both thermodynamic and kinetic constraints.

## Graphical abstract

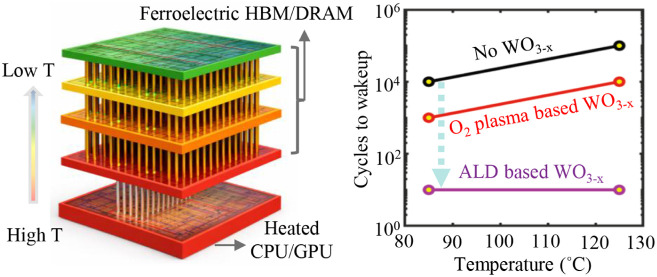

## Introduction

AI is fueling advances across domains such as high-performance computing, cloud infrastructure, mobile platforms, autonomous vehicles, and augmented reality, yet the massive data sets and complex models driving these applications are turning training and inference into critical memory and reliability challenges. Ferroelectric memory technologies, such as ferroelectric NAND (FE-NAND), ferroelectric random access memory (Fe-RAM), and ferroelectric field-effect transistors (FeFETs), have become leading contenders for nonvolatile memory solutions across various segments of the memory hierarchy.^1–3^ Recently, significant advancements in ferroelectric memories have been achieved, including the demonstration of a high-capacity dual-layer FE 1T-1C memory chip with a 32 Gb capacity and DRAM-comparable performance, as well as emerging three-dimensional integration of ferroelectric devices, exemplified by vertically stacked multilayer ferroelectric architectures for high-density integration.^4,5^ Despite advantages such as nonvolatility and high charge density, ferroelectric materials require a deeper understanding to ensure reliable operation, particularly under elevated temperatures.^6^

As the semiconductor industry advances toward 3D-IC integration, the intermediate step of memory-on-logic has demonstrated benefits comparable to those of a full technology node improvement. This memory-on-logic configuration shortens the communication distance between logic and memory, delivering up to 22% higher performance and 36% lower power consumption.^7^ However, thermal management poses a critical challenge. With an increasing number of stacked dies, tiers located farther from the heat sink and closer to heat-generating logic layers experience substantial thermal buildup due to thermal resistance and crosstalk^8^ (Figure 1a). Since logic dies are generally capable to operate at 105–125°C,^9^ memories must also be qualified to operate reliably at 125°C according to the JEDEC JESD22-A108 standard for 3D-IC qualification. Figure 1b summarizes the maximum operating temperature specifications of Micron DRAMs across different application scenarios.^10^ These specifications highlight the need for robust high-temperature performance, which is essential for enabling reliable integration of ferroelectric memories into emerging heterogeneous and monolithic 3D (H3D and M3D) systems and memory-on-logic architectures. Unfortunately, HfO_2_-based ferroelectrics, which form the foundation of many next-generation memory devices, remain highly vulnerable to degradation at elevated temperatures. It is well-established that increasing temperature induces an orthorhombic-to-tetragonal phase transition in ferroelectric materials, leading to pinching of the polarization loops and a gradual decrease in remnant polarization.^11–17^ Consequently, ferroelectricity can be lost under thermal stress, severely limiting device reliability.^16,18–20^ Although there have been lots of studies done on improving wakeup behavior and orthorhombic phase enhancement at room temperature using strategies like different atomic layer deposition (ALD) techniques,^21^ electrode engineering,^22–25^ controlling oxygen flow during deposition,^26^ high-temperature cycling,^27^ and interface engineering,^28–30^ systematic efforts to improve wakeup effect and pristine state polarization at elevated temperatures remain scarce in the literature.^31^

**Figure 1:**
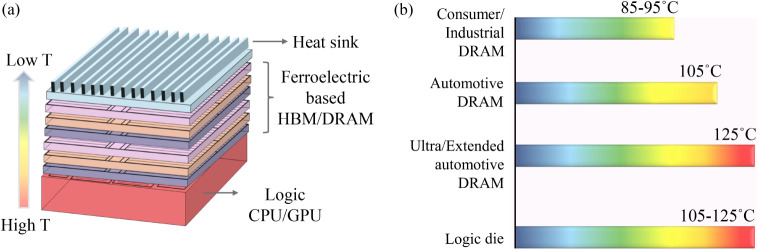
Importance of high-temperature performance enhancement of ferroelectric memories. (a) Schematic of 3D integration of memory on a logic architecture. Memories away from the heat sink get heated due to the heated logic die. (b) Operating temperatures of Micron DRAMs based on different applications.

Among various strategies to enhance ferroelectric device performance, interface engineering has shown particular promise. Several interfacial layers have been explored to improve endurance, leakage, and polarization characteristics. For example, NbO_2_ and TiO_2_ have been used for endurance enhancement,^32–34^ Pt has been employed to reduce leakage and wake-up effects,^26^ and TiON and WN_*x*_ have been investigated for boosting polarization.^29,35,36^ More recently, WS_2_ has demonstrated improvements in both polarization and endurance.^37^ In particular, WO_*x*_ has gained attention as an effective oxygen reservoir for improving different properties of ferroelectric capacitors at room temperature.^21,28,38–45^ However, its potential for enhancing ferroelectric performance at elevated temperatures remains largely unexplored.^46–48^

In this study, we demonstrate that incorporating WO_3–*x*_ as an interfacial oxygen reservoir layer significantly enhances the high-temperature performance of Hf_0.5_Zr_0.5_O (HZO)-based capacitors. WO_3–*x*_ is introduced at the interface between the bottom electrode and the HZO film, either by oxidizing the electrode via the O_2_ plasma treatment or through atomic layer deposition (ALD). This interface engineering approach increases the ability to retain the orthorhombic phase in HZO, suppressing the emergence of antiferroelectric behavior in pristine devices under thermal stress. As a result, the number of bipolar cycles required to achieve wake-up is significantly reduced in WO_3–*x*_-incorporated devices. First-principles density functional theory (DFT) calculations indicate that the presence of WO_3_ stabilizes the ferroelectric o-phase at elevated temperatures through a combination of effects: an increased tetragonal–orthorhombic phase energy gap in the Helmholtz free energy landscape and enhanced *X*_2_′ phonon mode coupling. These effects arise from the lower entropy and lattice anisotropy induced by WO_3_, which together favor the orthorhombic Pca2_1_ phase formation under both thermodynamic and kinetic considerations. This stabilization of ferroelectric behavior under thermal stress enhances the temperature resilience of the device. Our results provide a promising pathway for achieving thermally robust ferroelectric memories, such as Fe-RAMs and Fe-FETs, enabling their deployment in emerging 3D memory-on-logic architectures where elevated temperatures present a significant reliability challenge.

## Results and Discussion

### Experiment

Figure 2a illustrates the fabrication process flow of ferroelectric capacitors incorporating WO_3–*x*_ as an oxygen reservoir layer introduced via two distinct methods. In the first approach, partial oxidation of the sputtered W bottom electrode on Si was carried out using an O_2_ plasma treatment within the atomic layer deposition (ALD) chamber, resulting in the formation of an approximately 4 nm thick WO_3–*x*_ layer at the W surface. In the second approach, a plasma-enhanced ALD (PE-ALD) technique was employed to directly deposit 5 and 6 nm thick WO_3–*x*_ layers on the W electrode. For the reference device, the HZO layer was deposited directly on the bottom W electrode without any intermediate WO_3–*x*_ layer. Further details of the fabrication procedure are described in the Methods section.

**Figure 2:**
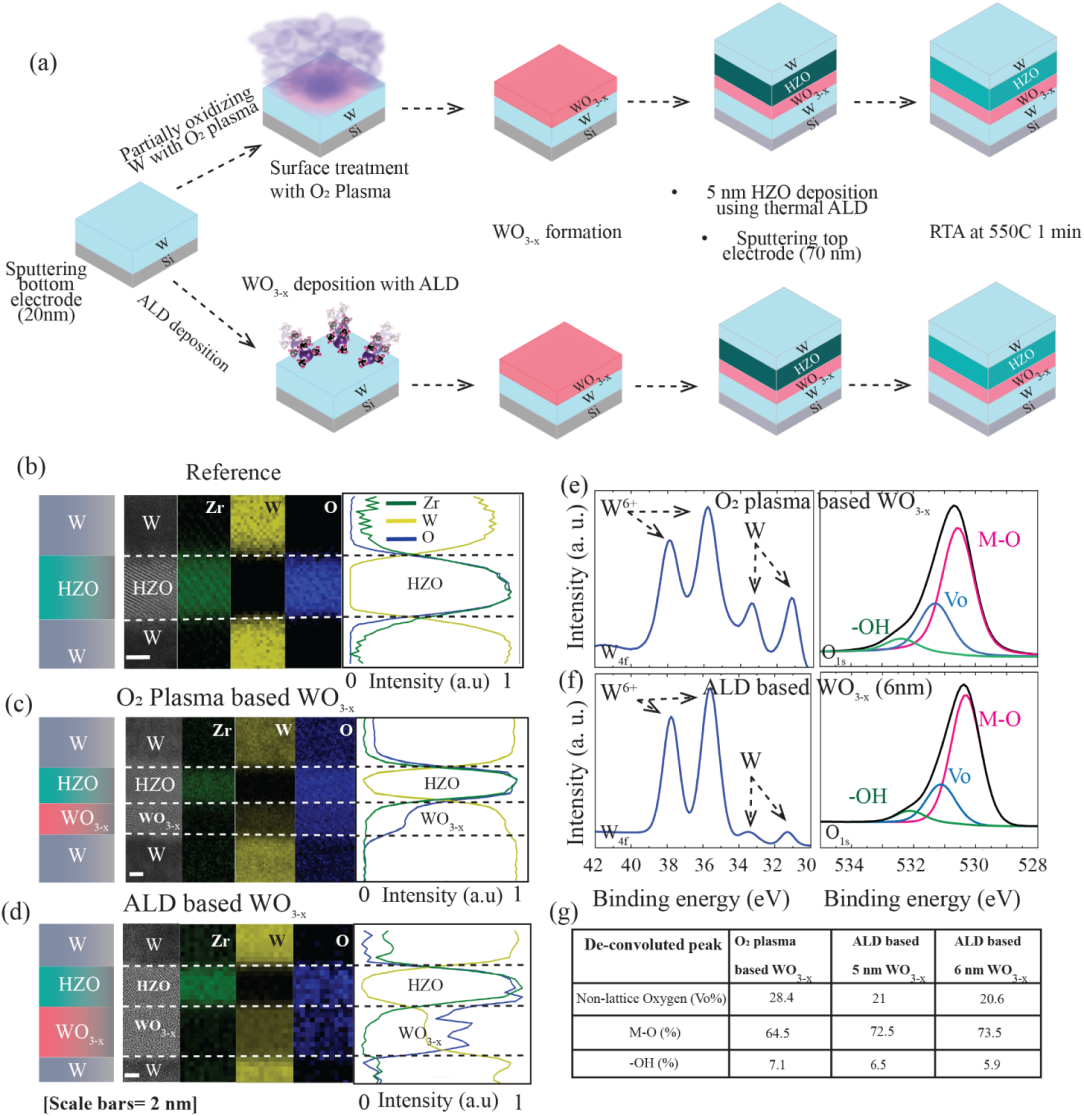
Device structures and material characterization of ferroelectric capacitors. (a) Fabrication process of O_2_ plasma- and ALD-based WO_3–*x*_ devices. (b–d) Cross-sectional STEM images of (b) reference and (c) O_2_ plasma- and (d) ALD-based 6 nm WO_3–*x*_ devices along with their EDS characterization. Material-count map coming from Zr (green), W (yellow), and O (blue) and the corresponding line scans for (b) reference, (c) O_2_ plasma, and (d) ALD devices. The line scans on the right of each material count map further confirm the layers and their interfaces. XPS spectra obtained from W 4f and O 1s orbitals of WO_3–*x*_ from (e) O_2_ plasma- and (f) ALD-based 6 nm WO_3–*x*_ samples. (e,f) Magenta, blue, and green curves in the O 1s plot are the deconvoluted peaks corresponding to M–O, nonlattice oxygen (*V*_o_), and −OH, respectively. (g) Table showing the percentage obtained from the deconvoluted peaks of the O 1s scan of different samples.

Scanning transmission electron microscopy (STEM) images of the cross-section of the fabricated devices are presented in the leftmost panel of Figure 2b–d, corresponding to the reference and O_2_ plasma- and ALD-based WO_3–*x*_ device structures, respectively. To verify the formation of WO_3–*x*_ at the bottom interface in both the O_2_ plasma- and ALD-based devices, energy-dispersive X-ray spectroscopy (EDS) was carried out. Elemental mapping confirmed the presence of distinct W and O signals at the interface in these devices, validating the successful formation of a WO_3–*x*_ layer. In contrast, the reference device exhibited no such signals, indicating the absence of an interfacial WO_3–*x*_ layer.

To assess the stoichiometry of WO_3–*x*_ films synthesized via an O_2_ plasma treatment and atomic layer deposition (ALD), X-ray photoelectron spectroscopy (XPS) analysis was conducted, as shown in Figure 2e and f. The W 4f spectra from both samples exhibit prominent W^6+^ peaks, confirming the successful formation of WO_3–*x*_. The O 1s spectra were deconvoluted to quantify the contributions from lattice and nonlattice oxygen species, with the extracted component ratios summarized in Figure 2g. Notably, the O_2_ plasma-based WO_3–*x*_ film shows a significant contribution from nonlattice oxygen, corresponding to an oxygen vacancy (*V*_o_) concentration of approximately 28.4%. In contrast, the ALD-deposited WO_3–*x*_ films with 5 and 6 nm thicknesses exhibit a comparatively lower *V*_o_ concentration of 21%. The full XPS spectrum for 5 nm ALD-grown WO_3–*x*_ is provided in Figure S1. These results indicate that while both methods yield substoichiometric WO_3–*x*_, the plasma process of the O_2_ introduces a higher density of oxygen vacancies. Furthermore, these findings underscore that the deposition technique, rather than film thickness, plays a critical role in governing WO_3–*x*_ stoichiometry, which in turn significantly impacts the electrical behavior of the devices, as elaborated in Figure 3.

**Figure 3:**
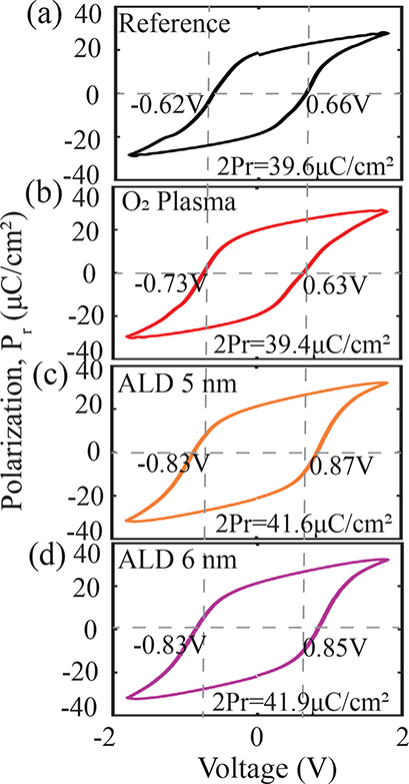
Electrical characterization at room temperature. *P*–*V* loops after 10^5^ cycles from (a) reference and (b) O_2_ plasma- and ALD-based (c) 5 nm and (d) 6 nm WO_3–*x*_ devices. 2P_r_ values are extracted from PUND measurement.

Figure 3 presents the polarization–voltage (*P*–*V*) characteristics of all fabricated devices, along with the extracted coercive voltage and remnant polarization values from *P*–*V* and positive-up negative-down (PUND) measurements, respectively. To decouple ferroelectric switching from leakage effects, PUND measurements were performed. All devices demonstrated comparable remnant polarization (2*P*_r_), confirming that the incorporation of WO_3–*x*_ does not compromise the ferroelectric polarization. Additionally, WO_3–*x*_ plays a beneficial role in enhancing device endurance, both at room and at higher temperatures, as shown in Figure S2 and supported by previous studies.^39,41^ A detailed analysis of temperature-dependent endurance improvements due to WO_3–*x*_ incorporation can be found in earlier work.^46^ Moreover, the inclusion of WO_3–*x*_ does not adversely affect the imprint and retention behavior, as illustrated in Figures S3 and S4.

The coercive voltage of the O_2_ plasma-based device closely matches that of the reference device, whereas the ALD-deposited WO_3–*x*_ devices exhibit a higher coercive voltage (Figure 3). This behavior indicates that the ALD-deposited WO_3–*x*_ is less conductive compared to the plasma-based counterpart, thereby dropping more voltage across the WO_3–*x*_ layer and requiring a higher switching voltage for the overlying HZO layer. This increase in the coercive voltage is consistent with the comparatively lower conductivity measured in these devices (Figure S5), and the lower oxygen vacancy (*V*_o_) concentration observed in the ALD samples from the XPS analysis in Figure 2e–g. Given that oxygen vacancies significantly affect the electrical conductivity of WO_3_ films,^45,49^ the reduced *V*_o_ content in ALD-deposited layers leads to decreased conductivity.

The polarization–voltage (*P*–*V*) and switching current–voltage (*I*_SW_–*V*) characteristics of all three devices in their pristine states were measured across a wide range of temperatures, as shown in Figure 4. At room temperature, both the reference and the O_2_ plasma-based WO_3–*x*_ devices exhibit similar *P*–*V* and *I*_SW_–*V* responses, while the ALD-deposited WO_3–*x*_ device shows marginally enhanced ferroelectric behavior (Figure 4a–c). Upon increasing the temperature to 85°C, the reference device that lacks any WO_3–*x*_ interfacial layer exhibits a pronounced antiferroelectric-like response, evidenced by a well-defined double-peak structure in the *I*_SW_–*V* curve (Figure 4d), indicating that the transition away from the orthorhombic ferroelectric phase initiates well before the targeted operating temperature (125°C) is reached.

**Figure 4:**
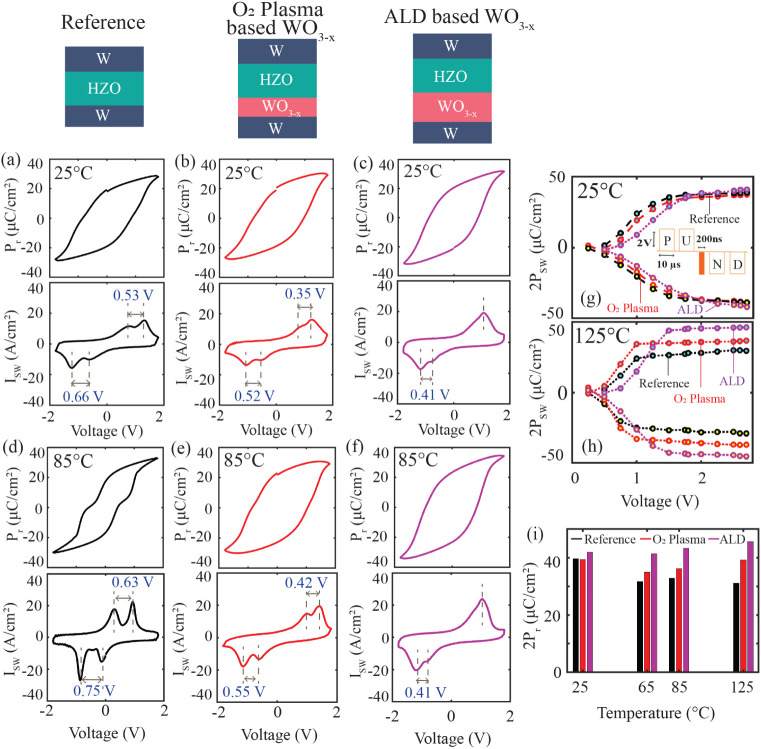
Temperature-dependent polarization and switching current characteristics at the pristine state. *P*–*V* and *I*_SW_–*V* characteristics at (a–c) 25 and (d–f) 85°C from (a,d) reference and (b,e) O_2_ plasma- and (c,f) ALD-based WO_3–*x*_ devices, respectively. (g,h) Polarization switched with 200 ns pulses at different voltages measured at 25 and 125°C, respectively, on pristine devices. (i) 2*P*_r_ obtained from PUND measurement with 2 V/10 μs square pulses at different temperatures in the pristine state.

In contrast, the O_2_ plasma-based device demonstrates improved thermal stability of ferroelectricity, evidenced by reduced separation of switching current peaks compared to the reference device at 85°C (Figure 4e). A smaller separation between same-direction switching peaks indicates reduced antiferroelectric-like behavior.^50^ Further enhancement in thermal robustness is observed for devices with ALD-based WO_3–*x*_ for the 6 nm layer (Figure 4f) and similarly for the 5 nm variant (Figure S6). These devices show a minor residual switching peak on the negative voltage side across all temperatures, suggesting that the WO_3–*x*_ incorporation effectively suppresses or delays the onset of the antiferroelectric phase transition.

This trend is validated by PUND measurements performed under varying temperature conditions in pristine states. Using 200 ns square pulses of varying amplitudes, the switched polarization (2P_SW_) was extracted. At room temperature, all devices exhibit comparable saturated polarization values under pristine conditions (Figure 4g). However, at 125°C, the reference device shows a significant drop in polarization relative to that of the WO_3–*x*_-containing devices (Figure 4h), indicating temperature-induced degradation of ferroelectricity in the absence of WO_3–*x*_. This observation is further reinforced by long-pulse (10 μs/2 V) PUND measurements across a wide temperature range on pristine devices, where the extracted 2*P*_r_ values confirm that ferroelectric properties deteriorate significantly in the reference device, while devices with WO_3–*x*_ maintain polarization more effectively (Figure 4i). These results highlight the crucial role of WO_3–*x*_ in stabilizing the ferroelectric orthorhombic phase at elevated temperatures in the pristine state, thereby preserving ferroelectric properties and suppressing unwanted phase transitions.

To confirm the stabilization of the ferroelectric o-phase with the incorporation of WO_3–*x*_ at elevated temperatures, grazing incidence X-ray diffraction (GI-XRD) measurements of the HZO layer in reference and O_2_ plasma- and ALD-based WO_3–*x*_ samples were performed at 25, 85, and 125°C. As shown in Figure 5a, the major o-(111)/t-(101) diffraction peak through Gaussian fitting from the samples having WO_3–*x*_ is shifted toward lower angles compared to the reference device, indicating a greater orthorhombic phase fraction. This observation aligns with the known peak positions of the o-(111) and t-(101) phases in HZO, located at approximately 30.4° and 30.8°, respectively. At 85°C, the peak separation between these samples decreases; however, the samples having WO_3–*x*_ still exhibit a discernible shift toward the orthorhombic phase, suggesting that a greater proportion of the orthorhombic phase is retained relative to the reference sample (Figure 5b). This behavior persists at 125°C, as shown in Figure 5c. The phase fractions of the o- and t-phases, extracted from the deconvolution of the o-(111)/t-(101) peaks measured at 125°C, are shown in Figure S7. The evolution of the peak positions with temperature for all of the samples is summarized in Figure 5d.

**Figure 5:**
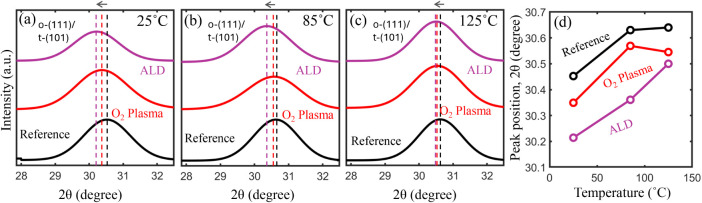
Grazing incident X-ray diffraction (GI-XRD) from the HZO film of reference and O_2_ plasma- and ALD-based 6 nm WO_3–*x*_ samples at (a) 25, (b) 85, and (c) 125°C. (d) Dominant peak (o-111/t-101) position of all the samples at different temperatures obtained from (a)–(c).

These results clearly demonstrate that the incorporation of WO_3–*x*_ helps maintain a higher orthorhombic phase fraction in HZO across a wide temperature range. And one of the reasons for this could be better lattice matching between orthorhombic Pca2_1_ HZO and monoclinic WO_3_ compared to that with W (Table S1). The monoclinic phase of WO_3_ is chosen for comparison, as this phase is confirmed by STEM imaging of the O_2_ plasma device (Figure S8). Additionally, the optimized *V*_o_ concentration in ALD-deposited WO_3–*x*_ is likely to promote enhanced o-phase stability in HZO compared to O_2_ plasma-based WO_3–*x*_, as prior studies have shown that stabilization of the o- phase in HZO is maximized within an optimal vacancy window in WO_3–*x*_, whereas both excessively vacancy-rich and near-stoichiometric (vacancy-poor) conditions lead to reduced o-phase content.^45,51^ The enhanced ferroelectric o-phase stability plays a critical role in suppressing the transition to antiferroelectric-like behavior at elevated temperatures, thereby contributing to improved ferroelectric performance.

The influence of electrical cycling on the switching behavior, with and without the presence of a WO_3–*x*_ interfacial layer at elevated temperatures, is studied in Figure 6. For the reference device, which lacks WO_3–*x*_, a significant number of 10^4^ cycles at 85°C and 10^5^ cycles at 125°C are required to suppress the characteristic double peaks in the *I*_SW_ response and achieve clean ferroelectric switching (Figure 6a,b). In contrast, the O_2_ plasma-based WO_3–*x*_ device exhibits stable ferroelectric switching behavior earlier than the reference, showing a single peak in the *I*_SW_ profile after just 10^3^ and 10^4^ cycles at 85 and 125°C, respectively (Figure 6c,d).

**Figure 6:**
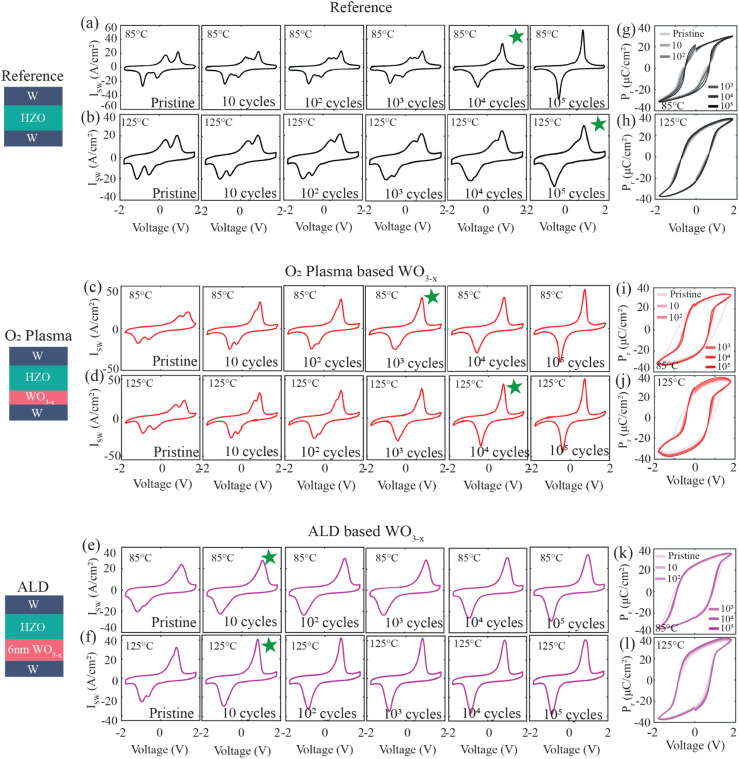
Temperature and cycling-dependent *P*–*V* and *I*_SW_–*V* characteristics. *I*_SW_–*V* characteristics at (a,c,e) 85 and (b,d,f) 125°C from pristine to 10^5^ cycles for (a,b) the reference, (c,d) the O_2_ plasma-based WO_3–*x*_, and (e,f) the ALD-based WO_3–*x*_ devices. Green asterisks denote the number of cycles required to remove the antiferroelectric-like double peak behavior. *P*–*V* loops from pristine to 10^5^ cycles at (g,i,k) 85 and (h,j,l) 125°C of (g,h) reference, (i,j) O_2_ plasma-based WO_3–*x*_, and (k,l) ALD-based WO_3–*x*_ devices.

Remarkably, for the ALD-based 6 nm WO_3–*x*_ device, as few as 10 cycles are sufficient for the transition to pure ferroelectric switching at both 85 and 125°C (Figure 6e,f). This phenomenon is true for the ALD-based 5 nm WO_3–*x*_ device as well (Figure S9). The substantial reduction in wake-up cycles observed in the ALD-based WO_3–*x*_ compared to those in the O_2_ plasma-based WO_3–*x*_ devices is consistent with the inherently gentler nature of the ALD process, which proceeds through self-limiting surface reactions and minimizes plasma-induced surface and interfacial defect generation. Collectively, these results underscore the critical role of the WO_3–*x*_ interfacial layer in enhancing the thermal and electrical stabilities of the ferroelectric phase. The corresponding *P*–*V* loops at 85 and 125°C after different cycling stages are shown in Figure 6(g–l) for the reference, O_2_ plasma and 6 nm ALD WO_3–*x*_ devices, and in Figure S10 for the 5 nm ALD WO_3–*x*_ device, further corroborating these trends. It is worth noting that once pure ferroelectric switching is achieved in the ALD-based WO_3–*x*_ devices at high temperatures after 10 bipolar cycles, it remains well preserved upon cooling to room temperature (Figure S11). Table 1 presents a benchmark of the 6 nm ALD WO_3–*x*_ device against prior studies of high-temperature ferroelectric capacitor behavior. The results indicate that the ALD WO_3–*x*_ device achieves superior pristine polarization and undergoes significantly fewer wake-up cycles during an elevated-temperature operation compared with recent reports. The 2*P*_r_ values of this work are obtained from the PUND measurement of Figure 4i.

**Table 1:** Comparison of Ferroelectric Properties at High Temperature (HT) with Prior Works

	[29]	[16]	[17]	[25]	[45]	This work
Film thickness (nm)	10	10	7	3	12	**5**
Write voltage (V)	3.5	2.2	2.1	1	3	**1.8**
Electric field (MV/cm)	3.5	2.2	3	3.3	2.5	**3.6**
Pristine 2*P*_r_ (μC/cm^2^)	26	15.1	16	5	32.94	**41.94**
Pristine 2*P*_r_ at HT (μC/cm^2^)	–	7 (120°C)	13.56 (125°C)	11.81 (85°C)	24.95 (100°C)	**45.57 (125°C)**
Cycles to wakeup at HT	10^3^ (100°C)	10^6^ (100°C)	10^4^ (100°C)	10^3^ (85°C)	10^6^ (100°C)	**10 (125°C)**

The leakage current density and trap generation rate during electrical cycling of all devices at different temperatures are presented in Figure S12. Although all the devices including the reference device show a similar trap generation rate at room temperature, WO_3–*x*_ containing devices show a significantly lower trap generation rate during electrical cycling at elevated temperatures, irrespective of the WO_3–*x*_ deposition method (Figure S12­(d–f)). This reduced trap generation is attributed to the oxygen-reservoir function of the WO_3–*x*_ layer, which contributes to the improved endurance (as shown in Figure S2) observed in these devices.^47^

### Density Functional Theory (DFT)

To investigate the origin of the stabilization of ferroelectric properties at elevated temperatures in the presence of WO_3–*x*_, density functional theory (DFT) calculations were performed to compare the stability of the orthorhombic Pca2_1_ phase in HZO when deposited on W and WO_3_. In this analysis, the atomic structure of bulk HZO was strained to match the lattice parameters of W and WO_3_, as illustrated in Table S1. Although the tungsten oxide layer in the fabricated ferroelectric capacitors is substoichiometric, the HZO lattice is strained to the WO_3_ lattice for the calculation, as the dominant feature in the W 4f XPS spectra corresponds to the W^6+^ oxidation state. Furthermore, WO_3–*x*_ containing oxygen vacancies does not exhibit a single well-defined periodic lattice constant due to the configurational disorder introduced by these vacancies. To account for oxygen deficiency, lattice mismatch calculations were carried out using a WO_3_ supercell containing a single oxygen vacancy, as summarized in Table S1. The mismatch values indicate that oxygen vacancies can tune the cell-averaged mismatch while primarily introducing local strain inhomogeneity.^52^ However, the key thermodynamic and kinetic trends discussed herein remain qualitatively unchanged.

Figure 7a presents the Helmholtz free energy of HZO in the monoclinic (m-), tetragonal (t-), and o- phases, each strained to match the lattice parameters of W and WO_3_, respectively. The yellow-shaded region indicates the high operating temperature range of interest (65–125°C). Within this range, the energy difference between the t- and o-phases of HZO strained to the WO_3_ lattice increases by approximately 20% compared to that strained to the W lattice, thereby favoring formation of the o-phase in the WO_3_ case. This behavior arises because W, having a cubic structure, induces a more cubic-like configuration in HZO, resulting in a higher entropy relative to the WO_3_ lattice (as reflected by the slope of the Helmholtz free energy curve). Consequently, the energy gap between the t- and o-phases is larger for WO_3_ at elevated temperatures. While W exhibits a greater lattice mismatch and thus higher surface energy than WO_3_, the increase in surface energies for both the o- and t-phases is comparable, yielding only a minor overall effect on the Helmholtz free energy.

**Figure 7:**
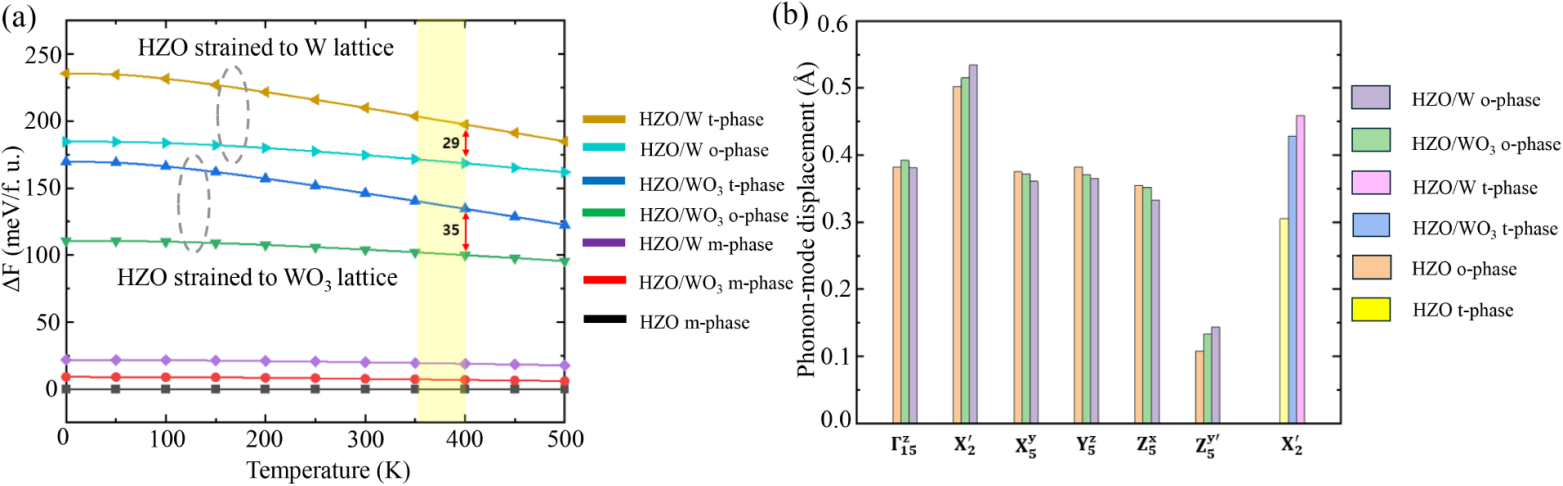
Density functional theory (DFT) analysis. (a) Helmholtz free energy difference (Δ*F*) for the 5 nm HZO film, relative to the m-phase. The yellow region and red arrow indicate the high-temperature operating range and free energy difference between t- and o-phases under W- and WO_3_-induced strain. (b) Phonon mode analysis under tensile strain induced by W and WO_3_, where the enhancement of the soft *X*_2_′ mode in the t-phase induces the phase transition to the o-phase.

Although the m-phase remains the thermodynamically most stable state even at a film thickness of 5 nm (i.e., exhibiting the lowest Helmholtz free energy), the kinetic barrier for the t → o phase transition is substantially lower than that for the t → m transition. Consequently, under kinetic constraints, the system preferentially transforms into the o-phase rather than the m-phase,^53^ in agreement with the calculations presented in Table S2. During cooling, the high-energy barrier similarly suppresses the formation of the m-phase, leading to the preferential development of the o-phase, which possesses a Helmholtz free energy lower than that of the t-phase. This trend is further supported by Figure 7a, where the o-phase remains more stable than the t-phase even at elevated operating temperatures, making it the energetically favorable configuration.

As shown in Figure 7b, the t-phase of HZO exhibits only the *X*_2_′ oxygen phonon mode, which drives the cubic-to-tetragonal phase transition. When strain is applied to match the lattice constant of WO_3_, corresponding to an approximately 1.7% increase in the lattice constant along the *x*-direction relative to the pristine structure, the *X*_2_′ phonon mode in the t-phase is enhanced. This enhancement brings its value closer to that of the o-phase, indicating that the t → o phase transition becomes more accessible. Under the W lattice, the larger lattice mismatch produces a greater amplitude of the *X*_2_′ mode compared to the WO_3_ case, leading, as shown in Table S2, to a lower t → o transition energy barrier. Overall, while the larger strain induced by W reduces the transition barrier relative to WO_3_, the o-phase in the WO_3_ case remains thermodynamically more stable than the t-phase due to the combined effects of surface energy at the 5 nm thick HZO and the large entropy associated with the larger in-plane lattice matched to the W cubic structure under nonequibiaxial strain (as described in Figure S13). These results are consistent with previous phase stabilization studies for the ZrO_2_–WO_3_ interface.^42^

The polarization switching barrier of HZO strained to the W and WO_3_ lattices was further calculated and found to be lower in the WO_3_-strained case (Figure S14). This difference arises because in the switching pathway the transition state corresponds to the t-phase, which exhibits a longer lattice constant along the *x*-direction than along the *y*-direction. For W, the structure is more cubic-like, with nearly identical lattice constants in both directions, leading to a higher switching barrier. In contrast, the anisotropy in the WO_3_-strained lattice reduces the barrier height. This energy barrier difference contributes to improved overall switching kinetics in devices incorporating WO_3_ or WO_3–*x*_ as the interfacial layer.

## Conclusion

In this study, we demonstrated the critical role of the WO_3–*x*_ interfacial layer in enhancing the thermal stability and ferroelectric performance of ultrathin Hf_0.5_Zr_0.5_O_2_ (HZO)-based capacitors. The incorporation of WO_3–*x*_, regardless of the deposition method and with carefully tuned oxygen vacancies, effectively suppressed the transition toward antiferroelectric-like behavior at elevated temperatures and enabled nearly wake-up-free operation with significantly fewer bipolar cycles. This improvement is attributed to the reduced relative Helmholtz energy for the orthorhombic phase in the presence of WO_3–*x*_, which favors the retention of ferroelectric properties, even under thermal stress. These findings offer a promising pathway toward the realization of robust ferroelectric memories suitable for 3D memory-on-logic architectures, where reliable high-temperature operation is essential.

## Methods

### Device Fabrication

Ferroelectric HZO capacitors were fabricated on p^+^ Si(100) substrates with a doping concentration of 10^20^ cm^–3^. A 20 nm W bottom electrode was deposited by using a Unifilm sputter system at a deposition rate of 200 Å. A 5 nm HZO layer was deposited using the Kurt J. Lesker ALD tool at 250°C using TDMAH and TDMAZ precursors for Hf and Zr, and H_2_O as the oxidant in the Th-ALD process,directly on W in the case of reference sample. For the O_2_ plasma sample, the W electrode was oxidized to form a WO_3–*x*_ interfacial layer through 10 cycles of oxygen plasma treatment in the ALD chamber prior to HZO deposition, ensuring no vacuum break. For the ALD WO_3–*x*_ samples, 20 and 30 cycles of ALD were used to deposit 5 and 6 nm WO_3–*x*_ layers, respectively, before depositing HZO in the Veeco Fiji G1 ALD system with a Bis­(*tert*-butylimino)­bis­(dimethylamino) (BTBMW) tungsten precursor and O_2_ plasma (300 W power, 40 sccm O_2_ flow, and 5 s plasma exposure per cycle) as the oxidant at 250°C. A 70 nm W top electrode was sputtered onto all devices, followed by rapid thermal annealing (RTA) at 550°C for 1 min in N_2_ atmosphere. Finally, standard photolithography and dry etching were used to define 50 μm × 50 μm capacitor structures.

### Energy-Dispersive X-ray Spectroscopy (EDS)

The EDS maps were collected using a Bruker E3DS detector with a 60 mm^2^ window on cross-sectional focused ion beam (FIB)-prepared samples in a Hitachi HD-2700 STEM. The spectra were then read via Hyperspy, and background subtractions were performed by selecting one window before and one window after the peaks of interest. Peaks corresponding to Zr–Kα, W-Lα, and O–Kα were used for mapping. To denoise the spectra, principal component analysis (PCA) was first applied, retaining four components, and then Online Robust Nonnegative Matrix Factorization (ORNMF) was performed using these four components identified. Line scans were created at the center of the horizontal direction (*x*-axis) for each map with a width of 40 pixels.

### X-ray Photoelectron Spectroscopy (XPS)

X-ray photoelectron spectroscopy (XPS) was conducted after depositing WO_3–*x*_ (either by ALD or using O_2_ plasma) on a sputtered bottom W electrode on a Si substrate. A Thermo K-alpha XPS system with an Al Kα X-ray source and a beam spot size of 200 μm was used for the scan. The flood gun was always on during data acquisition. XP emission peaks are charge-corrected to the carbon 1s peak which is set at 284.8 eV. XP emission backgrounds are subtracted with the Shirley algorithm, and the peaks are fitted with the Powell algorithm, with a convergence level <0.0001.

### Grazing Incidence X-ray Diffraction (GI-XRD)

After the removal of the top electrode of the devices using W etchant, GIXRD scan was done using a RIGAKU Smartlab XE diffractometer equipped with a Cu Kα source (40 kV, 50 mA) and a HyPix-3000HE detector. In-situ high-temperature data are collected on the RIGAKU Reactor X stage. Data were acquired in the range of 27°–34°, with an incidence angle of 0.5°, a scanning step of 0.04°, and a scanning speed of 0.05°/min. O and T peaks are constrained to have a Gaussian shape and the same fwhm during deconvolution.

### Electron Microscopy

Cross-sectional samples for scanning transmission electron microscopy (STEM) imaging and EDS were prepared using a Thermo Fisher Helios 5CX FIB/SEM equipped with a high-energy Focused Ion Beam (FIB) using Gallium-69 and operated at an accelerating voltage between 0.1 and 30 kV. The final polishing was performed at 10 pA and 2 kV. A Hitachi HD-2700 aberration- corrected STEM/SEM was used to capture STEM images, operated at a 200 kV accelerating voltage and a 27 mrad convergence semiangle. The spatial resolution was about 1.3 Å.

### Electrical Measurements

Electrical measurements were performed using a Cascade Microtech Summit 1200K semiautomated probe station, equipped with a Keysight B1500 semiconductor device analyzer. A 20 μs triangular pulse with an amplitude of 1.8 V was applied to measure the *P*–*V* and *I*_SW_ *–V* characteristics. For bipolar cycling, 200 ns pulses with an amplitude of 1.8 V were used. PUND measurements were conducted using 10 μs trapezoidal pulses, each with 10 μs rise and fall times. All measurements were done on 50 μm × 50 μm devices.

### Density Functional Theory (DFT)

Density functional theory (DFT) calculations were performed using the Vienna Ab initio Simulation Package (VASP).^54–57^ The electron–core interactions were described using the local density approximation (LDA)^58,59^ in conjunction with Blöchl’s projector augmented wave (PAW) method.^57,60^ A plane-wave cutoff energy of 500 eV was employed, and *k*-point meshes were sampled using the Monkhorst–Pack (MP)^61^ method with an 8 × 8 × 8 grid for conventional unit cells. Atomic positions were fully relaxed until the total energy and interatomic forces converged to less than 10^–8^ and 0.01 eV/Å, respectively. Vibrational free energies were obtained using the finite displacement method as implemented in Phonopy,^62^ which enabled accurate computation of the Helmholtz free energy. The surface energies were calculated using slab models comprising more than 8 layers with a vacuum region thicker than 15 Å. For slab calculations, the *k*-point meshes of the MP method with a 6 × 6 × 1 grid were used. The film thickness was set to 5 nm. The Helmholtz free energy *F* was calculated using the following relation:

$$F=U+F^{\mathrm{vib}}+\gamma \Omega$$

where *U* is the internal energy of the bulk system, *F* ^vib^ is the vibrational free energy, γ is the surface energy, and Ω is the surface area.

## Supplementary Material



## Notes

XPS of 5 nm ALD-based WO_3–*x*_; endurance; imprint; retention; conductivity; pristine *P*–*V* and *I*_SW_–*V* characteristics of 5 nm ALD-based WO_3–*x*_ device; deconvoluted GI-XRD peaks of 125°C measurement; lattice mismatch% of HZO with W, WO_3_, and single vacancy-contained WO_3_; STEM image of WO_3_; *I*_SW_–*V* and *P*–*V* of 5 nm ALD WO_3–*x*_ device at 85 and 125° C; *I*_SW_–*V* of 6 nm ALD-based WO_3–*x*_ device when cooled back to RT from 85 and 125° C; leakage and trap generation rate with cycling; energy barrier of HZO phase transition; vibrational entropies of W and WO_3_; switching barrier energy with and without WO_3_. (PDF)
