# Supplementary material for: ALD-Derived WO3–x Leads to Nearly Wake-Up-Free Ferroelectric Hf0.5Zr0.5O2 at Elevated Temperatures
Source: ACS Appl Electron Mater. 2026 Feb 4;8(4):1681–91. doi: 10.1021/acsaelm.5c02359 (PMC12937100; doi:10.1021/acsaelm.5c02359)
Supplement: Supplementary file 1 [file el5c02359_si_001.pdf]

Supporting Information

ALD-derived WO_{3-x} leads to nearly wake-up-free ferroelectric $Hf_{0.5}Zr_{0.5}O_2$ at elevated temperatures

Nashrah Afroze,[*,†,#] Jihoon Choi,[*,‡,#] Salma Soliman,[†] Chang Hoon Kim,[‡] Jiayi Chen,[†] Yu-Hsin Kuo,[†] Mengkun Tian,[¶] Chengyang Zhang,[†] Priyankka Gundlapudi Ravikumar,[†] Suman Datta,[†,§] Andrea Padovani,[‖] Jun Hee Lee,[*,‡,⊥] and Asif Khan[*,†,§]

†*Department of Electrical and Computer Engineering, Georgia Institute of Technology, Atlanta, GA-30332, USA.*

‡*School of Energy and Chemical Engineering, Ulsan National Institute of Science and Technology (UNIST), Ulsan-44919, South Korea.*

¶*Institute of Materials and Systems, Georgia Institute of Technology, GA-30332, USA.*

§*Department of Materials Science and Engineering, Georgia Institute of Technology, Atlanta, GA-30332, USA.*

‖*Department of Engineering Sciences and Methods (DISMI), University of Modena and Reggio Emilia, 42122 Reggio Emilia, Italy.*

⊥*Graduate School of Semiconductor Materials and Devices Engineering, Ulsan National Institute of Science and Technology (UNIST), Ulsan-44919, South Korea.*

#*These authors contributed equally to this work.*

E-mail: nafroze3@gatech.edu; jihoonchoi@unist.ac.kr; junhee@unist.ac.kr; akhan40@gatech.edu

Figure S1 shows the XPS spectra coming from 5nm ALD deposited $WO_{3\text{-}x}$ layer on W bottom electrode and Si substrate. W 4f spectrum shows dominant W^{6+} peaks confirmning the formation of $WO_{3\text{-}x}$. Deconvoluted peaks of O 1s spectrum are shown in Figure S1b.

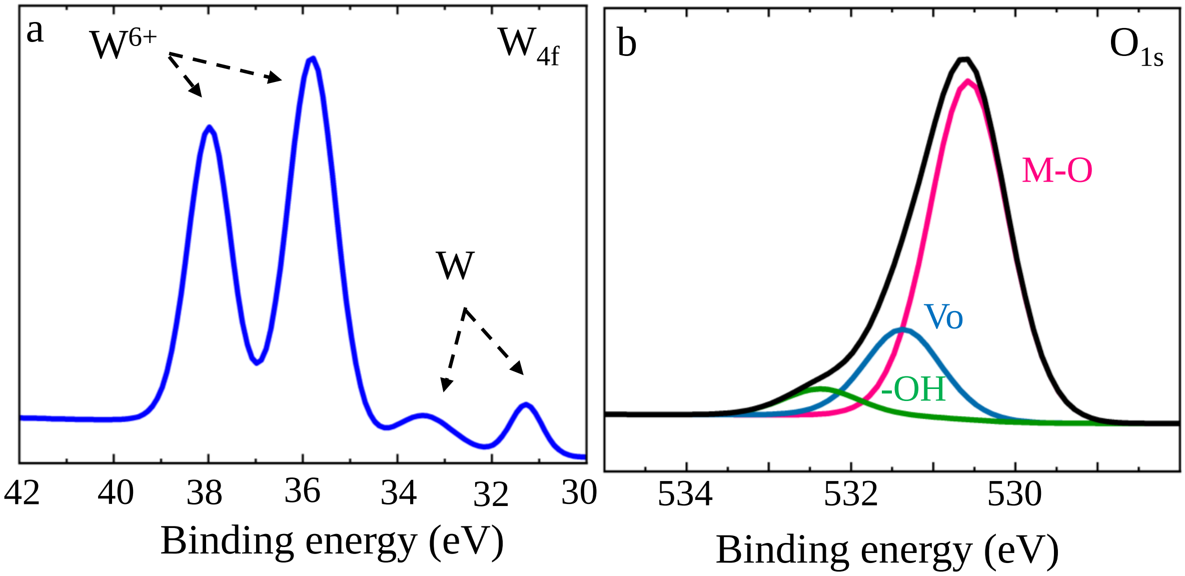

Figure S1: XPS spectra obtained from (a) W 4f and (b) O 1s orbitals of $WO_{3\text{-}x}$ from 5 nm ALD grown $WO_{3\text{-}x}$ samples. (b) Magenta, blue and green curves are the de-convoluted peaks corresponding to M-O, non-lattice Oxygen (V_o) and -OH respectively.

Figure S2a shows $2P_r$ versus cycles characteristics at room temperature when 200 ns bipolar pulses of 1.8 V were applied. The reference device lacking $WO_{3\text{-}x}$ has poor endurance compared to both O_2 plasma and ALD based $WO_{3\text{-}x}$ devices. $WO_{3\text{-}x}$ devices didn't break upto 10^{10} and 10^{11} cycles. The high-temperature endurance characteristics are presented in Figure S2b. Both the O_2 plasma and ALD WO_{3-x} devices exhibit higher endurance than the reference device over a wide temperature range. Although the ALD WO_{3-x} device reaches saturation polarization at 1.8 V (Figure 4g), endurance measurements were also performed using ±2 V bipolar pulses (orange curve) because of its slightly higher coercive voltage. At least three devices were measured for each condition.

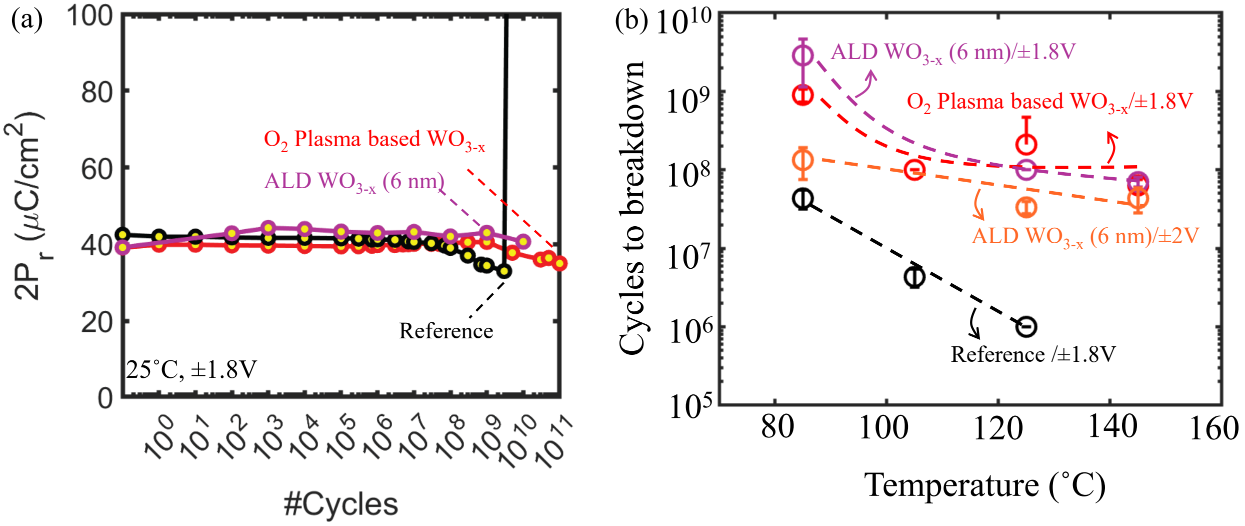

Figure S2: (a) $2P_r$ versus cycles characteristics with bipolar fatigue pulses of ±1.8 V/200 ns. PV measurements were also done at ±1.8 V. (b) Cycles to breakdown (endurance) at high temperatures. Both ±1.8 V and ±2 V bipolar cycling results are shown for ALD 6nm WO_{3-x} devices.

Figure S3 shows coercive voltage (V_c) versus cycles characteristics at 125°C when 200ns bipolar pulses of 1.8V were applied. Imprint doesn't degrade due to the presence of WO_{3-x} compared to reference device.

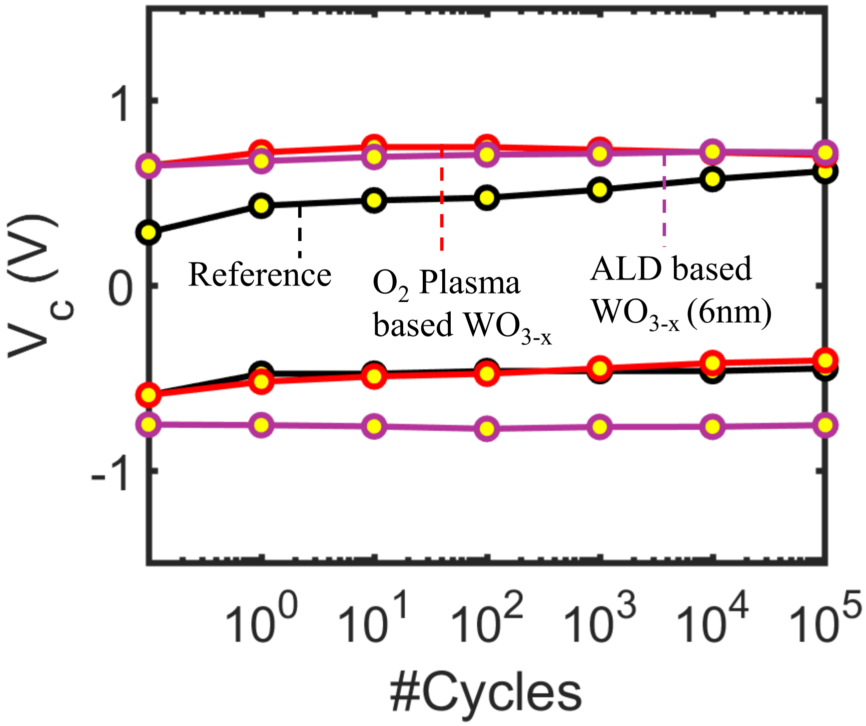

Figure S3: Shift in coercive voltage versus cycles at ±1.8V/200 ns , 125°C.

Figure S4 shows retention characteristics at 85°C by applying ±1.8 V square pulses. Retention doesn't degrade due to the presence of WO_{3-x} compared to reference device.

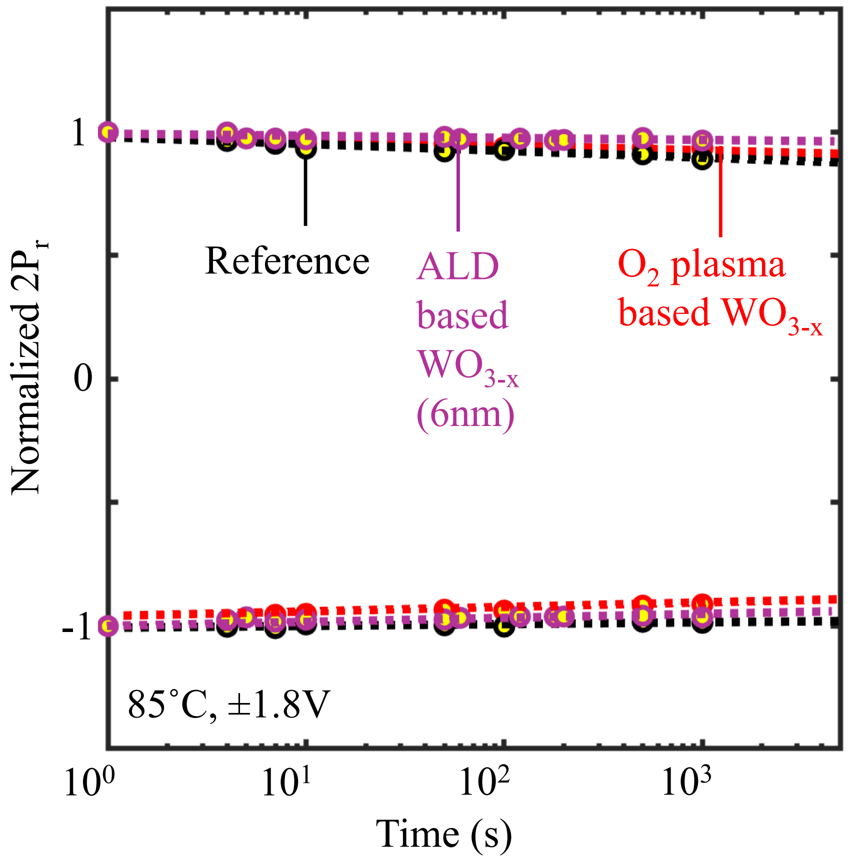

Figure S4: Retention characteristics at 85°C with ± 1.8 V.

Figure S5 shows conductivity of O_2 plasma and ALD based 6 nm $WO_{3\text{-}x}$ devices measured on 50μmx50μm devices by applying AC voltage of 100kHZ, 25 mV and DC sweep of 1.2 V.

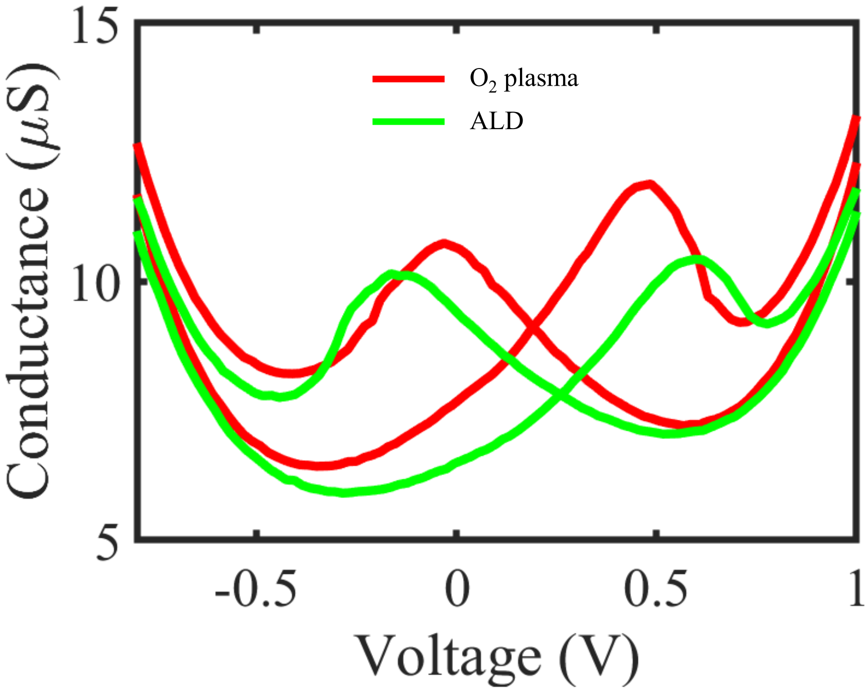

Figure S5: Conductivity versus applied voltage. O_2 plasma based WO_{3-x} device is more conductive compared to ALD based WO_{3-x} device.

Figure S6 shows the P-V and I_{SW}-V characteristics of pristine device measured at different temperatures from 5nm ALD deposited WO_{3-x} device. It shows minimal double peak characteristic (anti-ferro nature) like the 6nm WO_{3-x} device. These measurements were done by applying 1.8V/20μs bipolar triangular pulses.

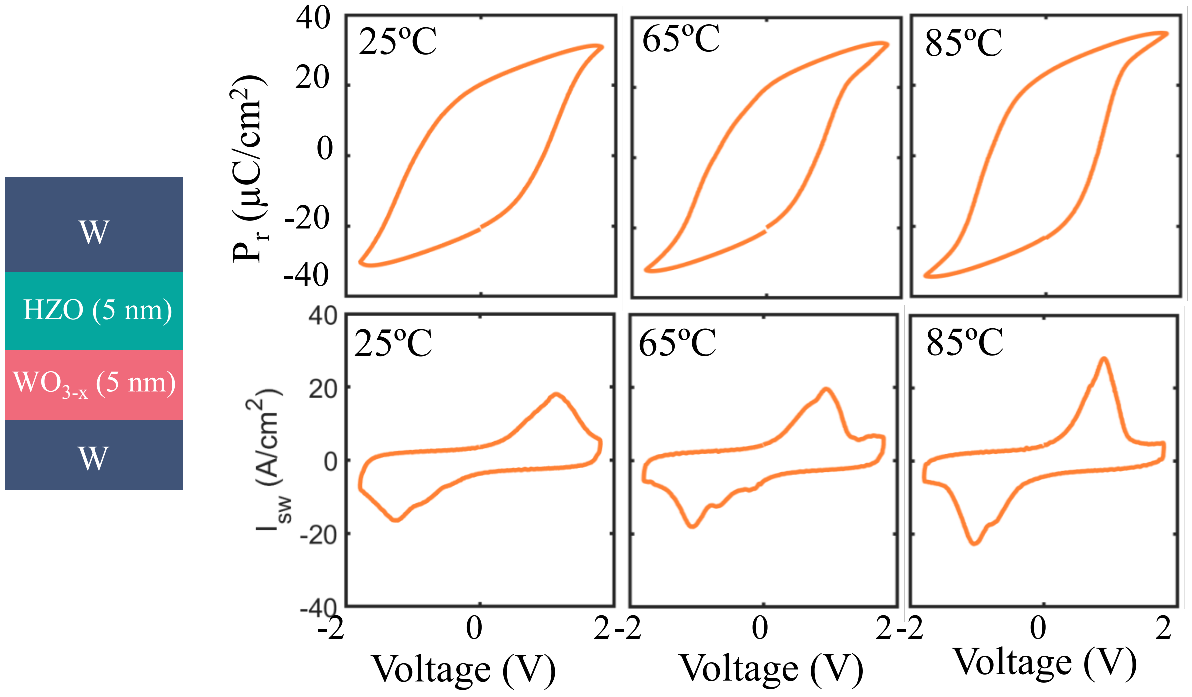

Figure S6: P-V and I_{SW}-V characteristics at 25, 65 and 85°C from pristine ALD deposited 5nm $WO_{3\text{-}x}$ device.

Figure S7 presents the deconvolution of the orthorhombic and tetragonal phase components from the Gaussian-fitted o-(111)/t-(101) diffraction peak measured from the HZO layer at 125°C. The ALD-deposited $WO_{3\text{-}x}$ sample exhibits the highest orthorhombic phase fraction (73%), whereas the reference sample shows the lowest orthorhombic phase content.

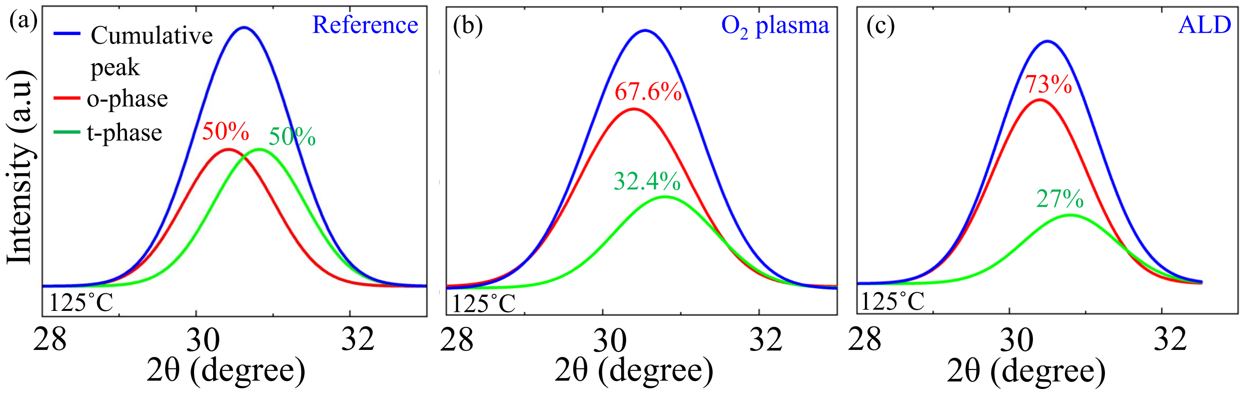

Figure S7: Deconvoluted o- (red) and t- (green) phase peaks from the fitted o-(111)/t-(101) diffraction peak (blue) of the HZO layer for (a) reference, (b) O_2 plasma, and (c) 6nm ALD-deposited $WO_{3\text{-}x}$ samples measured at 125 °C.

Table S1 shows lattice constant mismatch of HZO Pca2_1 (001) and (111) planes with W, WO_3 and 2x2x2 WO_3 supercell containing single Vo^{2+} respectively. The mismatch is lower with both WO_3 and $WO_{3\text{-}x}$ compared to W in both a and b directions. Supercell containing vacancy can tune the cell-averaged mismatch while primarily introducing local strain inhomogeneity.[1] Since HZO (111) plane exhibits a hexagonal geometry, the lattice was matched along the x-axis, and an equivalent magnitude of strain, corresponding to that applied along the x-axis, was imposed along the y-axis to introduce in-plane strain.

Table S1: Lattice mismatch (%) of HZO Pca2_1 orientations with W, WO_3 and WO_3 supercell containing single Vo^{2+}.

	W (a)	W (b)	WO_3 (a)	WO_3 (b)	$WO_3 - x$ (a)	$WO_3 - x$ (b)
HZO Pca2_1 (001)	1.76%	5.84%	1.40%	4.07%	1.21%	2.88%
HZO Pca2_1 (111)	2.56%		2.48%		2.24%	

Figure S8 shows the HAADF image of WO_3 layer of O_2 plasma device. Fast fourier transform (FFT) of the on-zone grain of WO_3 confirms it to be m-(001). Overlayed golden W atoms are coming from the crystal structure of monoclinic WO_3 obtained from .cif file of materials project.

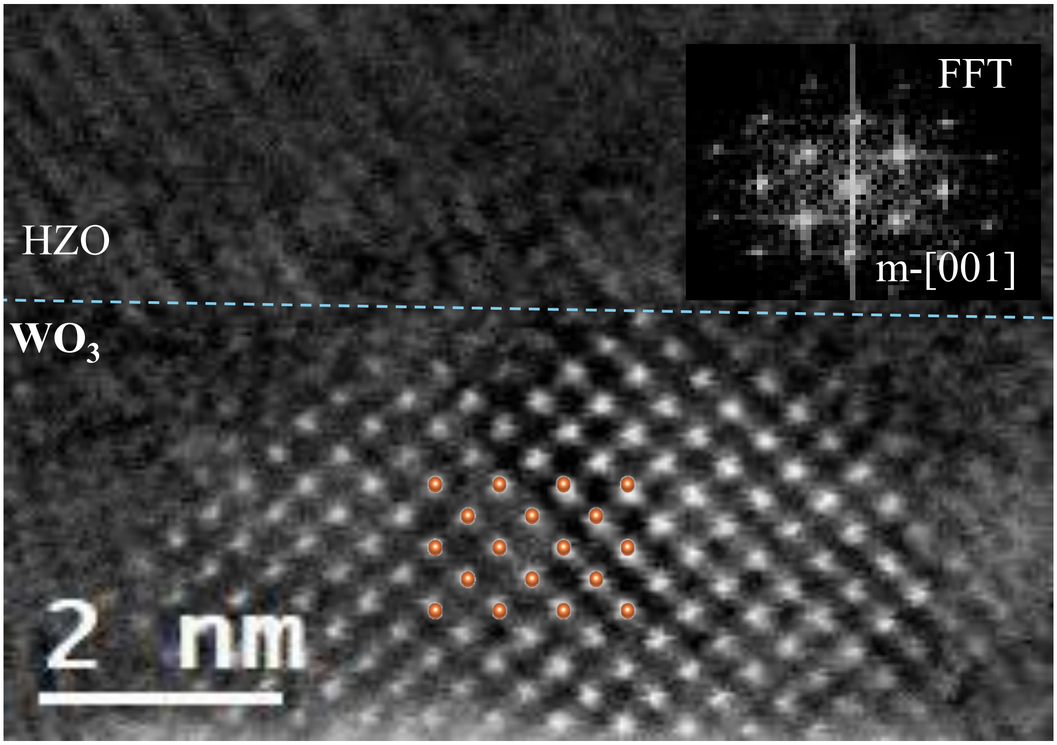

Figure S8: STEM image of WO_3 layer of O_2 plasma based device. Golden Overlayed W atoms are obtained from m-(001) crystal model. Fast Fourier Transform (FFT) of the WO_3 grain confirms it to be m-phase with [001] zone axis.

Figure S9 shows I_{SW}-V characteristics of 5nm ALD deposited $WO_{3\text{-}x}$ device measured at 85 and 125°C. Bipolar cycling was done by applying 200ns/1.8V pulses. Double peak in this device goes away just by applying 10 cycles, similar to 6nm ALD based $WO_{3\text{-}x}$ device. Much less cycles are needed for wakeup in devices having $WO_{3\text{-}x}$ compared to reference device irrespective of $WO_{3\text{-}x}$ thickness.

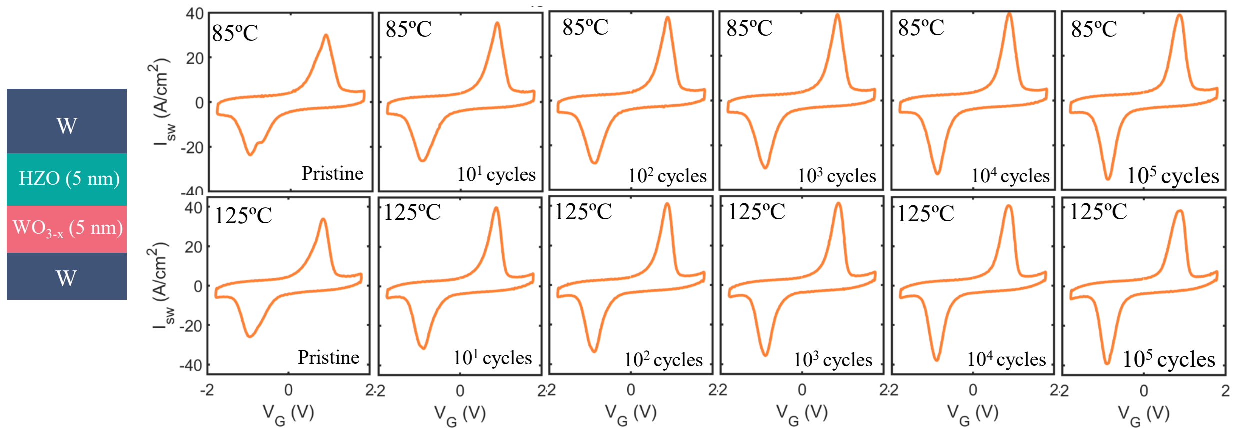

Figure S9: I_{SW}-V characteristics at 85 and 125°C from pristine to 10^5 cycles for ALD deposited 5nm $WO_{3\text{-}x}$ device.

Figure S10 shows P-V characteristics with cycling for 5nm ALD based $WO_{3\text{-}x}$ device measured at 85 and 125°C respectively. It shows better ferroelectric nature right from the pristine state compared to reference device as shown in Figure 4 (g-h).

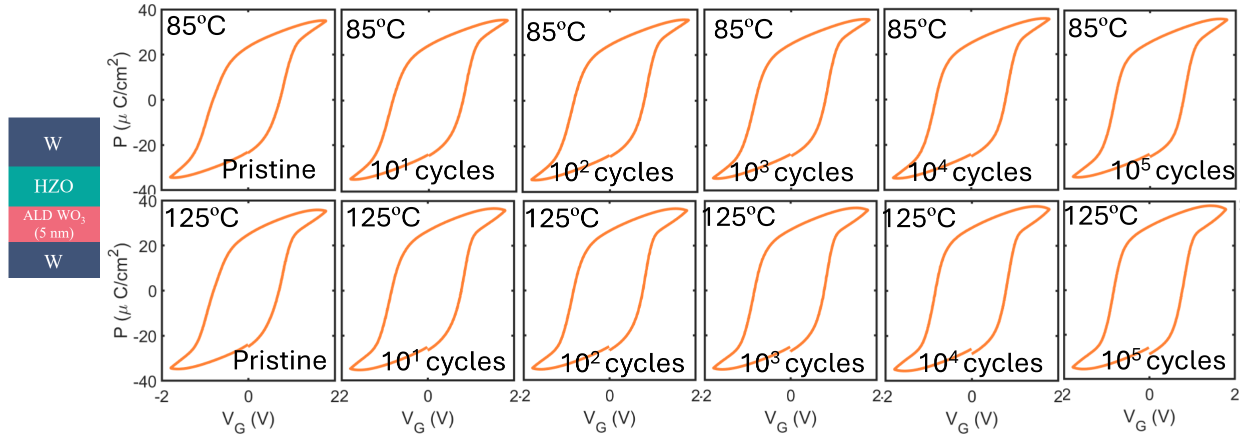

Figure S10: P-V characteristics at 85 and 125°C from pristine to 10^5 cycles for ALD 5nm $WO_{3\text{-}x}$ device.

When ten bipolar cycling pulses of ±1.8 V are applied to the ALD based 6 nm WO_{3-x} device at 85°C and 125°C, the pure ferroelectric switching established at elevated temperatures is well preserved upon cooling to room temperature, as shown in Figure S11.

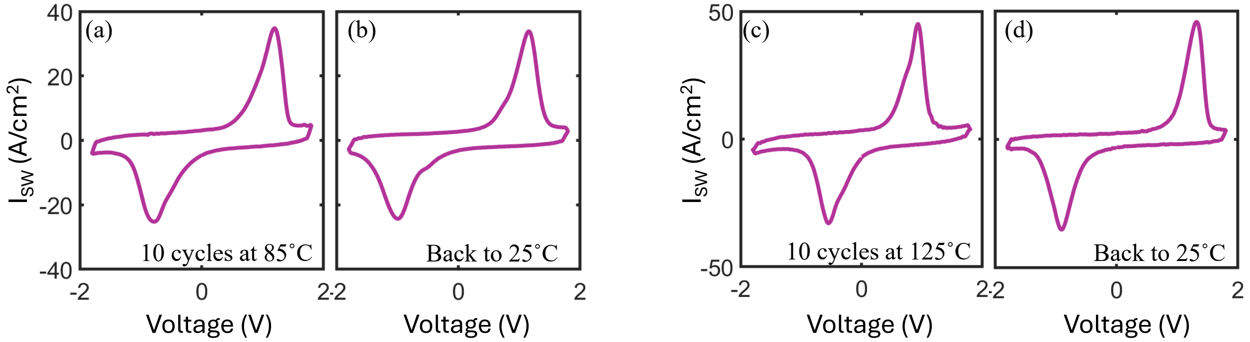

Figure S11: I_{SW} versus voltage characteristics of ALD 6nm WO_{3-x} devices (a) after applying 10 cycles at 85°C and (b) bringing back to 25°C, (c) after applying 10 cycles at 125°C and (d) bringing back to 25°C.

I-V characteristics are measured across a temperature range of 25°C to 125°C up to $5x10^6$ cycles, using ±1.7V, 200 ns write pulse trains for reference and O_2 plasma devices, and ±1.8V, 200 ns pulse trains for ALD devices. The measurements from pristine devices are shown in Figures S12 (a-c). Figures S12 (d-f) show the fractional increase of current density at cycle number = n with respect to the pristine state (n = 0), $\Delta J/Jo=(Jn-Jo)/Jo$, Jn being the current density at 1V at n-th cycle, as a function of cycle number. The slope of $\Delta J/Jo$ vs. cycles curves (which is a measure of trap generation rate with cycling) is similar at room temperature for all the devices. However, the slope is much lower in all the WO_{3-x} devices at elevated temperatures compared to the reference device.

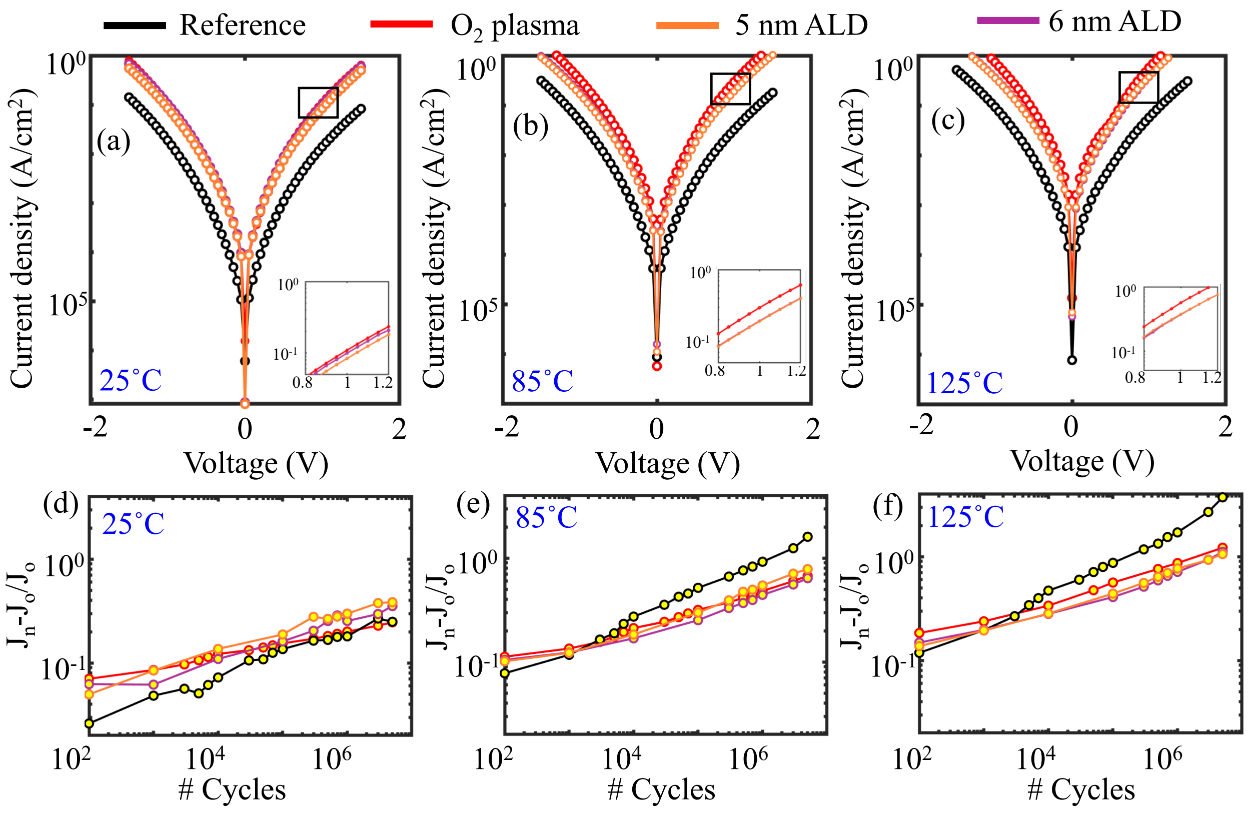

Figure S12: (a-c) Leakage current density versus voltage at pristine state at different temperatures. The insets show the region labeled by the black box. (d-f) ΔJ/Jo with cycling at 1V at (b) 25°C (c) 85°C and (d) 125°C.

Table S2 shows the energy barrier for t-to-m- and t-to-o- phase transition in HZO and HZO strained to W and WO_3 lattices. Transitioning to m- phase is energetically costly than to o- phase from t- phase in all cases.

Table S2: Calculated energy barriers for tetragonal-to-monoclinic (t-to-m) and tetragonal-to-orthorhombic (t-to-o) phase transitions.

	**Phase transition energy (meV)**	
	**t-to-m phase**	**t-to-o phase**
HZO	185	77
HZO/WO_3	176	73
HZO/W	188	69

Figure S13(a) presents the calculated vibrational entropies for the HZO structures matched

to the W and WO_3 lattices, respectively. The entropy of the W-matched system is consistently larger than that of the WO_3-matched case over the entire temperature range. Figure S13(b) shows the corresponding phonon DOS. As reported by Hua et. al.,[2] the longer interatomic distances lead to softer vibrations, and the softening of phonon modes—manifested as a shift of the phonon DOS toward lower frequencies—results in higher vibrational entropy, since low-frequency vibrations increase the number of accessible vibrational states at finite temperature. Similar behavior in Figure 7a originates from the larger in-plane lattice matched to the W cubic structure under non-equibiaxial strain, which induces softening of the low-frequency phonon modes and consequently increases the vibrational entropy.

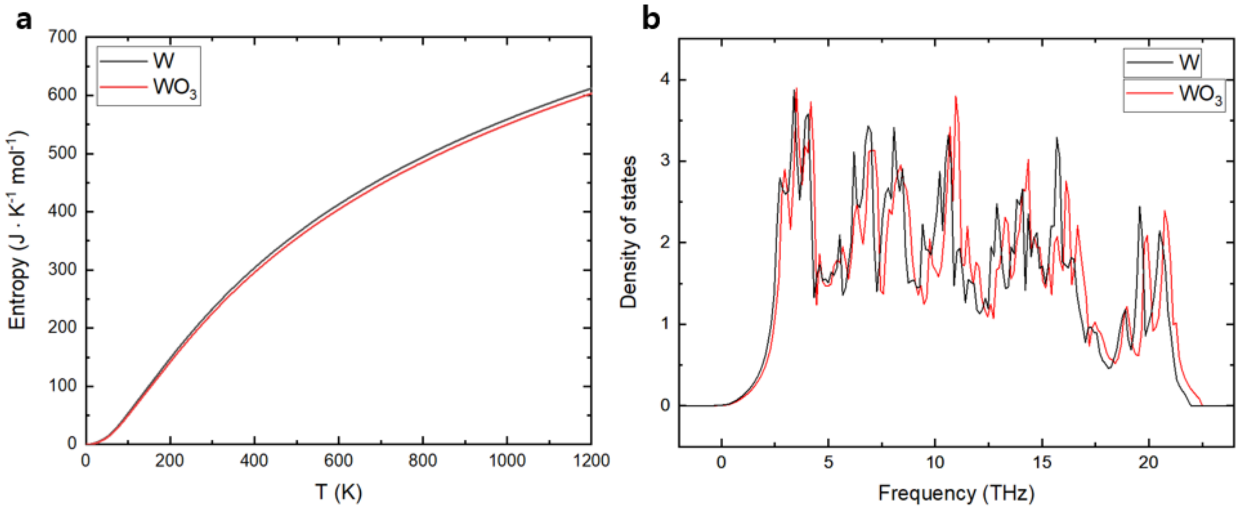

Figure S13: (a) Vibrational entropies for the HZO structures matched to the W and WO_3 lattices. (b) Corresponding phonon density of states (DOS).

In figure S14, it is clear that while transitioning from negative to positive polarization, the energy is higher at $\lambda = 0$ for HZO strained to W lattice compared to HZO strained to WO_3 lattice.

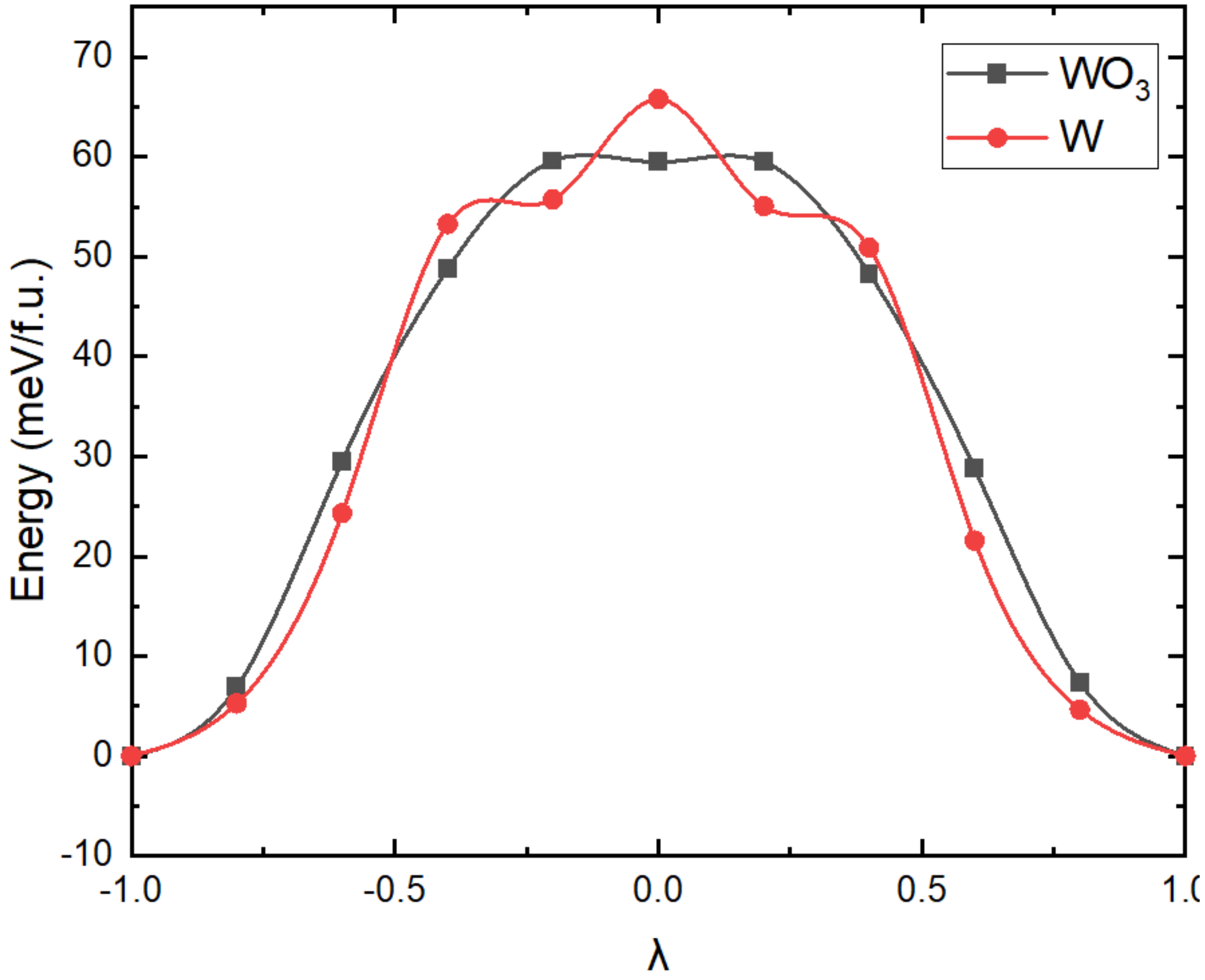

Figure S14: Ferroelectric switching pathways for HZO strained to WO_3 and W lattices. λ denotes the polar displacement, where $\lambda = -1$ corresponds to the ferroelectric (negative) state, $\lambda = 0$ to the paraelectric (zero) state, and $\lambda = 1$ to the ferroelectric (positive) state.

References

(1) Wang, W.; Janotti, A.; Van de Walle, C. G. Role of oxygen vacancies in crystalline WO 3. *Journal of Materials Chemistry C* **2016**, *4*, 6641–6648.

(2) Hua, X.; Hao, S.; Wolverton, C. First-principles study of vibrational entropy effects on the PbTe-SrTe phase diagram. *Phys. Rev. Mater.* **2018**, *2*, 095402.
